# P-95. Utilization of Cell-Free DNA Metagenomic Analysis for Early Detection and Microbial Identification in Prosthetic Joint Infections: A Prospective Cohort Study in Korea

**DOI:** 10.1093/ofid/ofaf695.324

**Published:** 2026-01-11

**Authors:** Jung Ah Lee, Dongju Won, Eun Hwa Lee, Jaehoon Kim, Seung-Tae Lee, Kwan Kyu Park, Saeam Shin, Su Jin Jeong

**Affiliations:** Yonsei University College of Medicine, Seoul, Seoul-t'ukpyolsi, Republic of Korea; Yonsei University College of Medicine, Seoul, Seoul-t'ukpyolsi, Republic of Korea; Yonsei University College of Medicine, Seoul, Seoul-t'ukpyolsi, Republic of Korea; Yonsei University College of Medicine, Seoul, Seoul-t'ukpyolsi, Republic of Korea; Yonsei University College of Medicine, Seoul, Seoul-t'ukpyolsi, Republic of Korea; Yonsei University College of Medicine, Seoul, Seoul-t'ukpyolsi, Republic of Korea; Yonsei University College of Medicine, Seoul, Seoul-t'ukpyolsi, Republic of Korea; Yonsei University College of Medicine, Seoul, Seoul-t'ukpyolsi, Republic of Korea

## Abstract

**Background:**

Prosthetic joint infection (PJI) is a severe complication of hip or knee arthroplasty, often necessitating invasive intervention and posing a high risk of adverse outcomes. Early diagnosis and tailored antibiotic therapy are critical for the effective management of PJI. This study evaluated the utility of cell-free deoxyribonucleic acid (cfDNA) extracted from synovial fluid to diagnose PJI and identify the causative pathogens.

**Figure 1.**
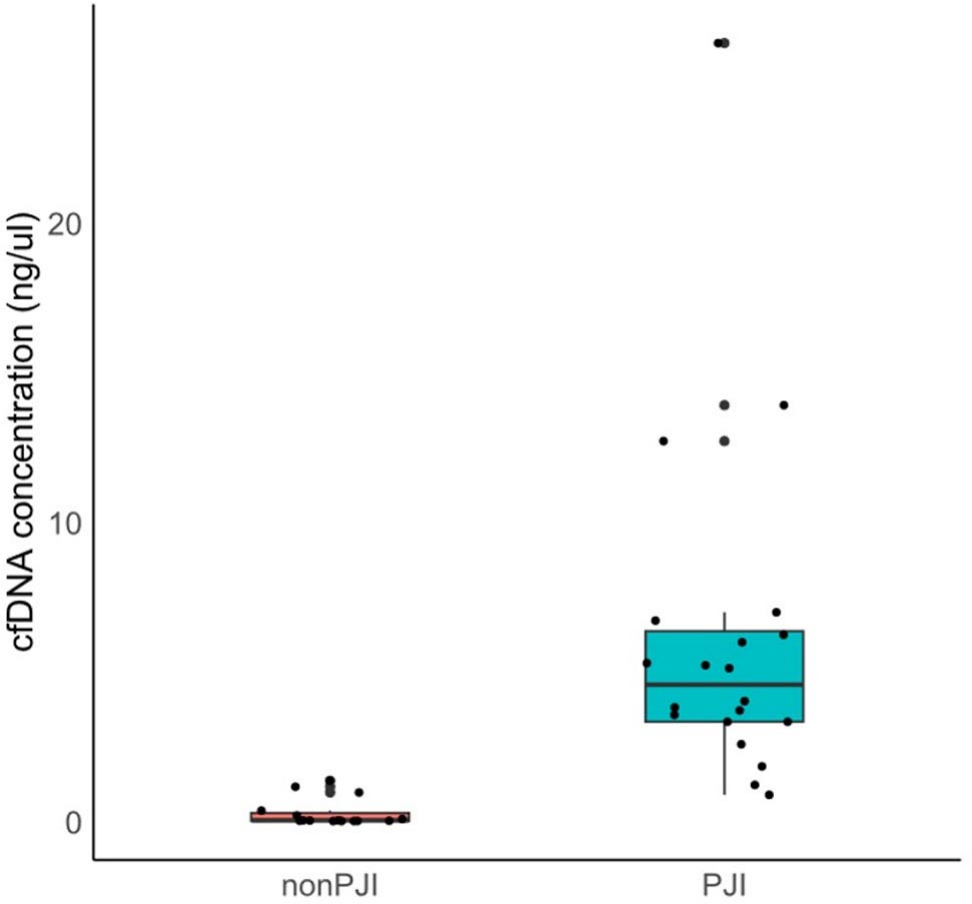
Comparison of Synovial Fluid cfDNA Concentration Between PJI and Non-PJI Groups

**Figure 2.**
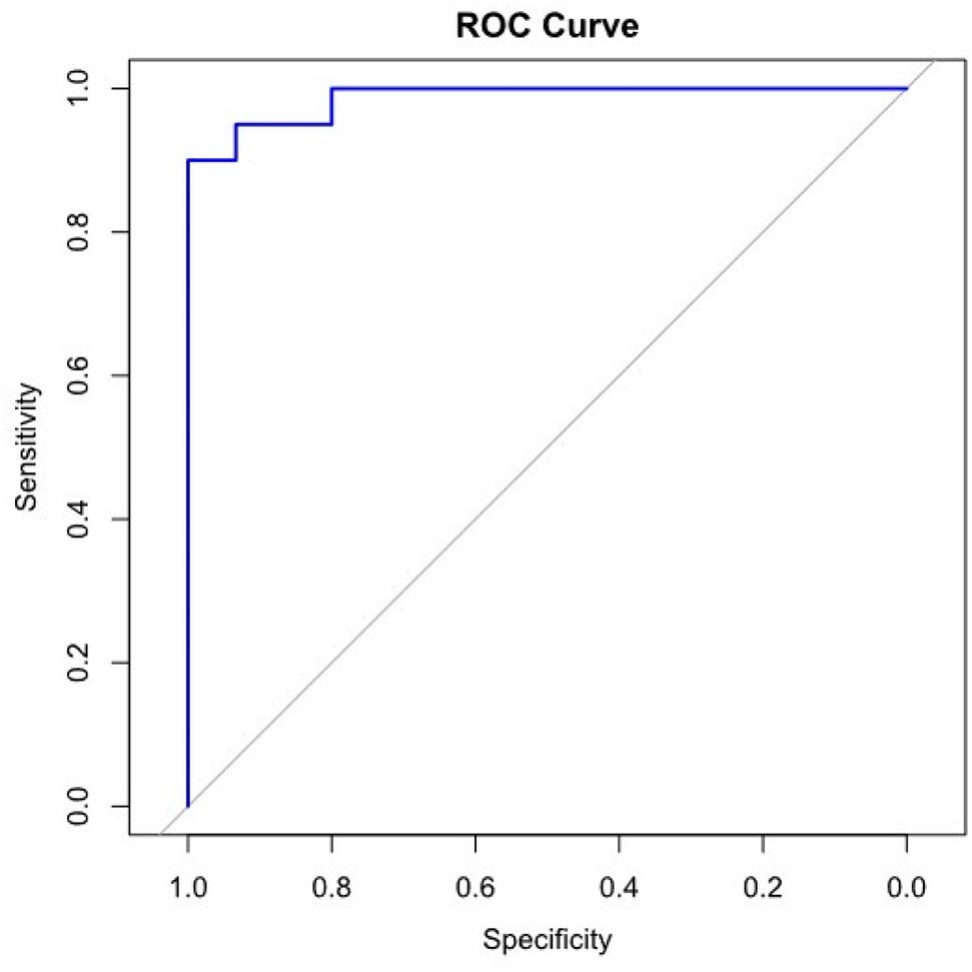
ROC curve for Determining the Optimal cfDNA Concentration Cut-Off for PJI diagnosis cfDNA concentration of 1.59 ng/ul or above indicates possibility of PJI. (sensitivity, 0.90; specificity, 1.00), respectively.cfDNA, cell-free deoxyribonucleic acid; PJI, prosthetic joint infection.

**Methods:**

This prospective, single-center study included a PJI group consisting of patients with confirmed infections based on the European Bone and Joint Infection Society criteria and a non-PJI group comprising patients without suspected PJIs who underwent joint surgery or aspiration. Synovial fluid samples were collected from all patients, and various culture methods, including conventional synovial fluid, sonication, and tissue and blood cultures, were applied along with cfDNA analysis.

**Figure:**
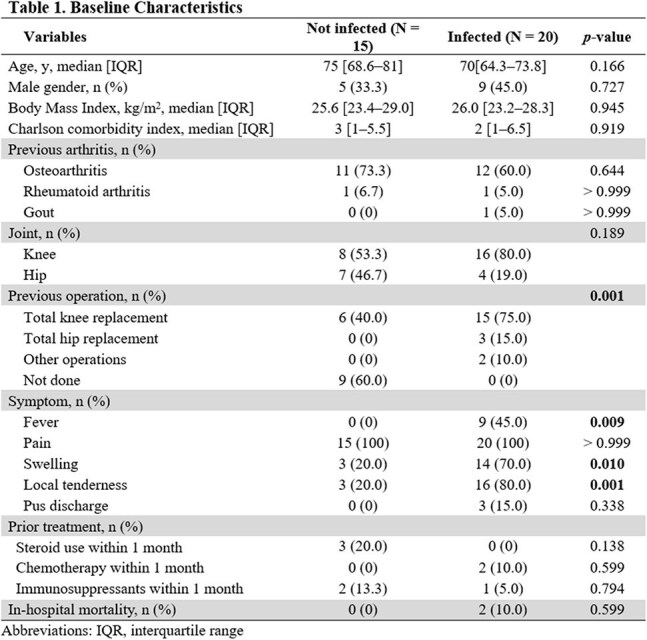

**Figure:**
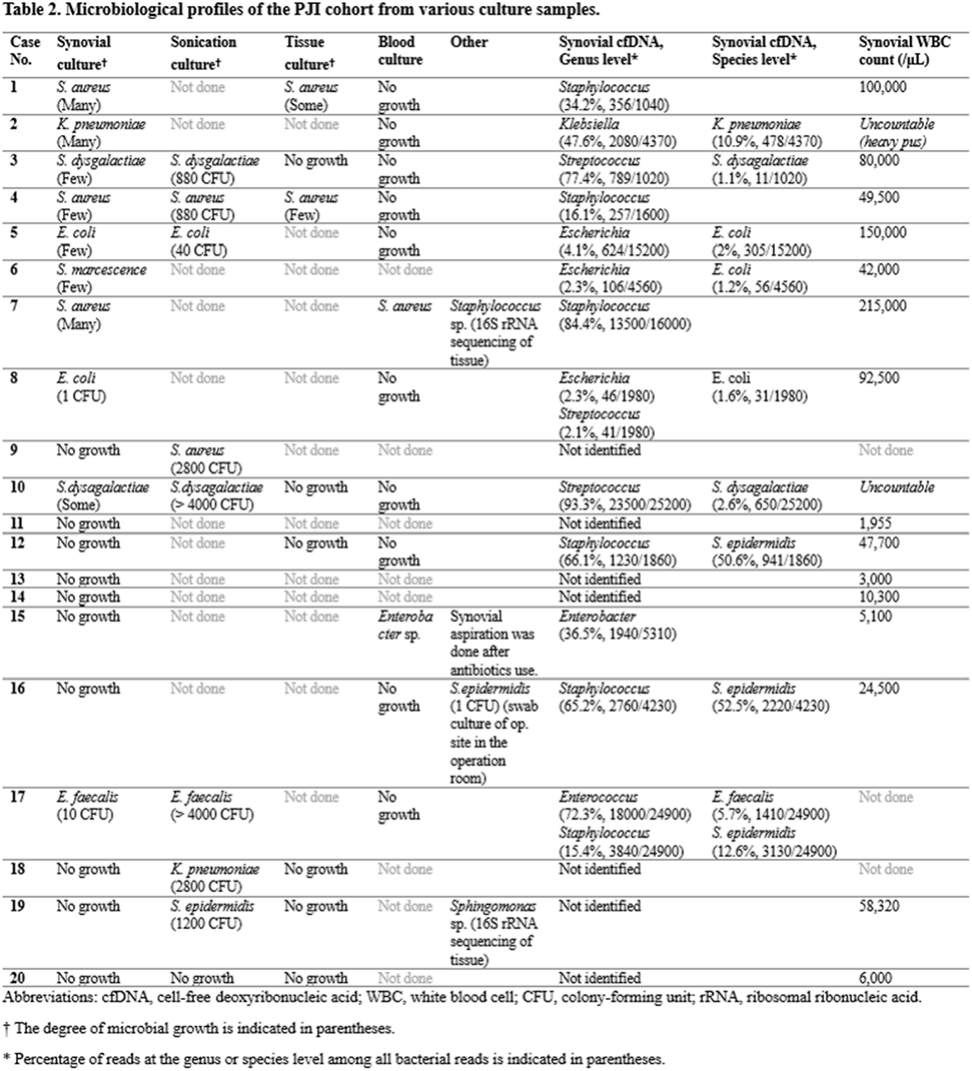

**Results:**

A total of 35 patients were included, with 20 diagnosed with PJI and 15 classified as non-PJI. The median cfDNA concentration in synovial fluid was significantly higher in the PJI group (4.560 ng/μl, interquartile range (IQR) [3.320–6.348]) compared with the non-PJI group (0.028 ng/μl, IQR [0.009–0.273]) (*p* < 0.001). The Youden index identified a cfDNA concentration ≥ 1.59 ng/μl as strong likelihood of PJI. Culture positivity rates in the PJI group were as follows: synovial culture (10/20, 50.0%), sonication culture (8/9, 88.9%), tissue culture (2/8, 25.0%), and blood culture (2/12, 16.7%). The bacterial detection rate of cfDNA was 65.0% (13/20).

**Conclusion:**

cfDNA concentration was significantly higher in the PJI group, with synovial cultures showing substantial agreement. Additionally, cfDNA sequencing detected pathogens after antibiotic treatment and identified multiple pathogens in polymicrobial infections. These findings highlight cfDNA analysis as a valuable diagnostic tool for PJI, with the potential to enhance current diagnostic approaches.

**Disclosures:**

All Authors: No reported disclosures

